# Editorial: Reproductive Barriers and Gene Introgression in Rice Species, Volume II

**DOI:** 10.3389/fpls.2022.974613

**Published:** 2022-07-22

**Authors:** Yohei Koide, Kazuki Matsubara, Dayun Tao, Kenneth L. McNally

**Affiliations:** ^1^Laboratory of Plant Breeding, Research Faculty of Agriculture, Hokkaido University, Sapporo, Japan; ^2^Institute of Crop Science, National Agriculture and Food Research Organization, Tsukuba, Japan; ^3^Yunnan Key Laboratory for Rice Genetic Improvement, Food Crops Research Institute, Yunnan Academy of Agricultural Sciences, Kunming, China; ^4^T. T. Chang Genetic Resources Center, International Rice Research Institute, Los Baños, Philippines

**Keywords:** breeding, evolutionary biology, genetic resource, genome variation, hybridization, introgression, reproductive barriers

Distant hybridization and introgression undoubtedly play a crucial role in Asian rice (*Oryza sativa* L.) domestication and diversification and will continue to play an important role as humans face to climate change challenges. The limited genetic variation within current rice varieties necessitates the use of various approaches to improve production under increasingly harsher and more frequent abiotic and biotic stresses resulting from climate change. The wild relatives of Asian cultivated rice represent valuable reservoirs of variation for both traditional breeding and advanced technology breeding such as *de novo* domestication, redomestication, and gene editing improvement.

To achieve the complex task mentioned above, the use of distant relatives of rice as donors of genetic diversity has been attempted to improve resistance together with productivity in rice varieties. However, reproductive barriers, which appear in prezygotic and/or postzygotic phases, are often observed in interspecific or intersubspecific crosses and prevent the introgression of useful genes. Therefore, reproductive barriers and gene introgression are major concerns in rice breeding, and are also of concern in rice evolutionary and developmental genetics.

This Research Topic is the second of a series meant to gather current knowledge in this field and share it with the scientific community to accelerate rice genetic improvement.

In this Research Topic, two articles described the phenotypic and genotypic nature of wild rice species. Eizenga et al. characterized the phenotypic variation of the *Oryza rufipogon* species complex (ORSC), the wild progenitor of Asian rice (*O. sativa* L.). The ORSC comprises perennial, annual, and intermediate forms historically designated as *O. rufipogon, O. nivara*, and *O. sativa* f. *spontanea*, respectively, based on non-standardized morphological, geographical, and/or ecological-based species definitions and boundaries. A collection of 240 diverse ORSC accessions, previously characterized by genotyping-by-sequencing (113,739 SNPs), was phenotyped for a total of 57 traits in three different locations. Combined with information from morphology-based and genetic identity-based species definition analyses, one phenotypic group contained predominantly *O. rufipogon* accessions characterized as perennial and largely out-crossing and one contains predominantly *O. nivara* accessions characterized as annual and largely self-crossing. The third group was identified as *Oryza* spp. and admixture levels of *O. sativa* accounted for more than 50% of the genome. Meanwhile, Hasan et al. discuss the issue of the two distinct taxa found growing together in northern Australia, *O. meridionalis* (including annual and perennial forms) and *O. rufipogon-*like taxa shown to have a chloroplast genome sequence closer to *O. meridionalis* than to *O. rufipogon* from Asia. A comparison of chloroplast and nuclear genome sequences indicated hybridization between these taxa. Individuals with intermediate morphology had high nuclear genome heterozygosity consistent with a hybrid origin. An examination of specific genes suggested that some wild plants were early generation hybrids.

For reproductive barriers, two articles reviewed the latest issues regarding hybrid sterility in rice. Zhang Y. et al. reviewed systematically the relationship between hybrid sterility and divergence of Asian cultivated rice, finding that more than 40 conserved and specific loci have been shown responsible for hybrid sterility of subgroup crosses. Most studies have focused on the sterility barriers between indica and japonica crosses, ignoring hybrid sterility among other subgroup crosses, leading to neither a systematic understanding of hybrid sterility and subgroup divergence nor an effective understanding of how to use the strong heterosis between subgroups in Asian cultivated rice. Future studies will aim to create a blueprint to identify intraspecific hybrid sterility loci for (1) overcoming the intraspecific hybrid sterility according to the parent subgroup type identification, (2) allowing the use of heterosis among subgroups, and (3) unlocking the relationships between hybrid sterility and Asian cultivated rice divergence. Myint and Koide reviewed the distribution of hybrid sterility genes, from the viewpoint of transmission ratio distortion in male (*m*TRD), female (*f* TRD), or *si*TRD (sex-independent transmission ratio distortion). Among 49 hybrid sterility loci surveyed, the number of loci for *m*TRD is the largest (33), in contrast to that of *f* TRD (10) and *si*TRD (7). Additionally, the sterility based on gamete specificity is distributed disproportionately between interspecific and intraspecific hybrids. This bias in the frequency of sex-specificity in the TRD system might reflect different evolutionary pressures acting on the *Oryza* system, suggesting that they are not driven only by mutation and genetic drift.

Identification of genes for reproductive barriers is also ongoing. Soe et al. reported their research progress regarding rice hybrid weakness, which was observed in F_1_ hybrids. Genetic analysis for low temperature-dependent hybrid weakness was conducted in a rice F_2_ population derived from Taichung 65 (T65, *japonica*) and Lijiang-Xin-Tuan-Heigu (LTH, *japonica*). They observed that growth at 24°C enhanced hybrid weakness, whereas recovery occurred at 34°C. Two major QTLs were detected on chromosome 1, named *hybrid weakness j 1* (*hwj1*), and on chromosome 11, termed *hybrid weakness j 2* (*hwj2*). Further genotyping indicated that the hybrid weakness was due to an incompatible interaction between the T65 allele of *hwj1* and the LTH allele of *hwj2*.

Kubo et al., using a map-based cloning strategy, found that *HWE1* and *HWE2* encode the Esa1-associated factor 6 (EAF6) protein, a component of histone acetyltransferase complexes. The indica *hwe1* and japonica *hwe2* alleles lacked functional EAF6, demonstrating that double recessive homozygote causes hybrid breakdown in rice. These findings suggest that EAF6 plays a pivotal role in transcriptional regulation of essential genes during the vegetative and reproductive development of rice.

Zhang C. et al. undertook fine mapping and characterization of two loci (*TRD4.1* and *TRD4.2*) for transmission ratio distortion (TRD) using large F_2_ segregating populations. The two loci exhibited a preferential transmission of the ZS97 alleles in the derived progeny. Reciprocal crossing experiments using near-isogenic lines harboring three different alleles at *TRD4.1* suggested that male gametic selection occurred. Moreover, the transmission bias of *TRD4.2* was diminished in heterozygotes when they carried homozygous *TRD*4.1^ZS97^. These findings broaden the understanding of the genetic mechanisms of TRD and offer an approach to overcome the barrier of gene flow between the groups of varietal types in rice.

Chen et al. reported their preliminary results using RNAi and *OsMYB76R* as a reporter to efficiently create and verify gametophytic male sterility in rice. Guo et al. reported their research on the development of wide-compatible indica lines by pyramiding multiple neutral alleles of indica–japonica hybrid sterility loci. The results showed that wide-compatible indica lines (WCILs) by pyramiding multiple neutral (n) alleles of five loci showed wide compatibility with both indica and japonica rice varieties. Therefore, the WCILs can be used to develop intraspecific indica–japonica hybrid rice with normal fertility.

For gene introgression from the wild relatives of Asian cultivated rice, Li et al. reported preliminary results of the genetic network underlying rhizome development in *O. longistaminata*. Beerelli et al. mapped *qTGW8.1* to a 2.6 Mb region in all three generations with PV 6.1 to 9.8%. This stable and consistent *qTGW8.1* allele from *O. nivara* can be finely mapped for the identification of causal genes for higher grain weight.

Zhang B. et al. reviewed recent progress on the development of introgression lines (ILs). They classified rice improvement into five generations: semi-dwarf rice, intra-subspecific hybrid rice, inter-subspecific introgression rice, and inter-subspecific indica-japonica hybrid rice. They found that indica-japonica hybrid sterility was mainly controlled by six loci. The indica-japonica hybrid sterility can be overcome by developing indica-compatible japonica lines (ICJLs) or WCILs using genes at the six hybrid sterility loci. With the understanding of the genetic and molecular basis of indica-japonica hybrid sterility and the development of molecular breeding technology, the development of indica-japonica hybrid rice has become possible.

The fine pieces of research collected in this topic facilitated a comprehensive understanding of issues in the introgression of traits of interest from the wild genetic resources to breeding programs in rice. We believe that this platform for enhancing exchange and promoting development has merit to be continued with other aspects such as gametophytes gene and non-random recombination of independent genes.

## Author contributions

All authors listed have made a substantial, direct, and intellectual contribution to the work and approved it for publication.

## Conflict of interest

The authors declare that the research was conducted in the absence of any commercial or financial relationships that could be construed as a potential conflict of interest.

## Publisher's Note

All claims expressed in this article are solely those of the authors and do not necessarily represent those of their affiliated organizations, or those of the publisher, the editors and the reviewers. Any product that may be evaluated in this article, or claim that may be made by its manufacturer, is not guaranteed or endorsed by the publisher.

